# Supplementary material for: Predicting clinical outcomes from large scale cancer genomic profiles with deep survival models
Source: Sci Rep. 2017 Sep 15;7:11707. doi: 10.1038/s41598-017-11817-6 (PMC5601479; doi:10.1038/s41598-017-11817-6)
Supplement: Supplementary file 1 — Supplementary Information [file 41598_2017_11817_MOESM1_ESM.doc]

**SUPPLEMENTARY INFORMATION**

**Predicting clinical outcomes from large scale cancer genomic profiles with deep survival models**

Safoora Yousefi^1^, Fatemeh Amrollahi^1^, Mohamed Amgad^1^, Chengliang Dong^2^, Joshua E. Lewis^3^, Congzheng Song^4^, David A Gutman^5^, Sameer H. Halani^6^, Jose Enrique Velazquez Vega^7^, Daniel J Brat^7,8^, *Lee AD Cooper^1,3,8^

*Correspondence to lee.cooper@emory.edu

^1^Department of Biomedical Informatics, Emory University School of Medicine, Atlanta, GA 30322

^2^Department of Biostatistics, Mailman School of Public Health, Columbia University, New York, NY 10032

^3^Department of Biomedical Engineering, Georgia Institute of Technology / Emory University School of Medicine, Atlanta, GA 30322

^4^Department of Computer Science, Cornell University, Ithaca, NY 14850

^5^Department of Neurology, Emory University School of Medicine, Atlanta, GA 30322

^6^Emory University School of Medicine, Atlanta, GA 30322

^7^Department of Pathology and Laboratory Medicine, Emory University School of Medicine, Atlanta, GA 30322

^8^Winship Cancer Institute, Emory University, Atlanta, GA 30322

Table S1A. Summary of integrated feature set datasets.

| Dataset | Histology / subtype | Samples | Median survival  (deceased) | Median follow up  (right censored) | Proportion censored (%) |
| --- | --- | --- | --- | --- | --- |
| BRCA | Breast invasive carcinoma | 524 | 1053 | 928.5 | 90.5 |
| GBMLGG | Glioma | 560 | 480 | 566 | 64.1 |
|  | IDH wild-type  astrocytoma | 203 | 375.5 | 294 | 32.0 |
|  | oligodendroglioma | 145 | 947.5 | 651 | 87.6 |
|  | IDH mutant  astrocytoma | 209 | 1262 | 608.5 | 79.4 |
| KIPAN | Kidney Carcinoma | 641 | 793 | 1360.5 | 72 |
|  | Renal cell chromophobe (KICH) | 43 | 855 | 56 | 88.4 |
|  | Renal clear cell (KIRC) | 407 | 822 | 1488 | 64.8 |
|  | Renal papillary cell (KIRP) | 191 | 624 | 761 | 84.8 |
| OV | Ovarian serous cystoadenocarcinoma | 206 | 1201 | 1284.5 | 30 |
| UCEC | Uterine Corpus Endometrial Carcinoma | 194 | 726 | 1106 | 86 |

Table S1B. Summary of transcriptional feature set datasets.

| Dataset | Histology / subtype | Samples | Median survival  (deceased) | Median follow up  (right censored) | Proportion censored (%) |
| --- | --- | --- | --- | --- | --- |
| BRCA | Breast invasive carcinoma | 1092 | 1272 | 761 | 86 |
| GBMLGG | Glioma | 692 | 510 | 608 | 62 |
|  | IDH wild-type astrocytoma | 239 | 383 | 415 | 29 |
|  | oligodendroglioma | 171 | 962 | 682 | 87 |
|  | IDH mutant / astrocytoma | 267 | 1246.5 | 685 | 77 |
| KIPAN | Kidney Carcinoma | 887 | 787.5 | 1213 | 74 |
| OV | Ovarian serous cystoadenocarcinoma | 306 | 1082 | 761 | 40 |
| UCEC | Uterine Corpus Endometrial Carcinoma | 370 | 714 | 1098 | 84 |

**Table S2. Description of features (see Excel file).**

**Table S3. C-index values from Monte Carlo validation (see Excel file).**

**Table S4. Feature risk scores for glioma models (see Excel file).**

**Table S5. Feature risk scores for transfer learning experiments (see Excel file).**
